# Mad Honey Poisoning Presenting With Anaphylaxis and Cardiovascular Instability: A Case Series

**DOI:** 10.1155/carm/1634503

**Published:** 2026-04-05

**Authors:** Abal Baral, Kusha K. C.

**Affiliations:** ^1^ Department of Internal Medicine, Jiri Hospital, Dolakha, Nepal; ^2^ Department of Internal Medicine, Pashupati Chaulagain Memorial Hospital, Dolakha, Nepal

**Keywords:** anaphylaxis, case series, grayanotoxin, mad honey, wild honey

## Abstract

**Background:**

Mad honey differs from commercial honey due to contamination with grayanotoxins. These toxins are produced when honey bees extract nectar and pollen from various *Rhododendron* species. While cardiovascular toxicity is well‐documented, anaphylactic reactions are rare and underreported.

**Case Presentation:**

This case series describes seven patients presenting to a rural primary care hospital in Nepal between June 2024 and January 2025. Patients were males aged 36–66 years who consumed 5–25 mL of wild honey. The clinical presentation varied: five patients presented with classic symptoms of dizziness, vomiting, bradycardia, and hypotension. Notably, two patients presented with features of anaphylaxis (angioedema, urticaria, and respiratory distress) without initial bradycardia. All patients improved within 24–48 h under supportive management, which included atropine for bradycardia and intramuscular adrenaline (1:1000 concentration) for anaphylaxis.

**Conclusion:**

Mad honey poisoning can present with a spectrum of symptoms ranging from cholinergic toxicity to anaphylaxis. This series highlights the diagnostic challenge of consuming wild honey contaminated with grayanotoxins and underscores the need for clinicians to recognize anaphylaxis as a potential, albeit less common, manifestation.

## 1. Introduction

Mad honey differs from commercial honey due to contamination with grayanotoxins [1]. Grayanotoxins are polyhydroxylated cyclic hydrocarbons devoid of nitrogen [2]. These toxins are produced when honey bees extract nectar and pollen from various *Rhododendron* species [3]. The traditional practice of hunting and consuming wild honey in the Himalayan regions significantly raises the risk of grayanotoxin exposure [4].

While mad honey intoxication is a global phenomenon reported across Asia, Europe, and the Americas [1, 3], it remains a significant public health issue in Nepal. Locally, it is used in alternative medicine for treating hypertension, diabetes, gastrointestinal disorders, sexual dysfunction, and various other ailments [1, 5, 6].

The literature primarily focuses on the cardiovascular effects of grayanotoxins, specifically bradycardia and hypotension. However, hypersensitivity reactions remain an uncommon and poorly characterized presentation. Herein, we present a case series of seven patients with mad honey poisoning managed in a rural primary care hospital. Uniquely, this series details two cases presenting with anaphylaxis, contributing to the limited evidence on immunological responses to grayanotoxin‐contaminated honey.

## 2. Case Discussion

### 2.1. Case 1

A 36‐year‐old male presented to the emergency department (ED) with shortness of breath, abdominal cramps, and vomiting following the recreational ingestion of 15 mL of wild honey. He had no significant medical history. On admission, he was drowsy and hypotensive (80/60 mmHg) with significant bradycardia (48 bpm). Electrocardiogram (ECG) confirmed sinus bradycardia. He was managed with intravenous (IV) fluid resuscitation, atropine (2 mg), and supportive care. Laboratory investigations were unremarkable. His hemodynamic parameters stabilized within 24 h, and a repeat ECG revealed a normal sinus rhythm prior to discharge.

### 2.2. Case 2

A 39‐year‐old male with a history of hypertension presented with vomiting and dizziness after consuming 10 mL of wild honey to control his blood pressure. On admission, he was agitated but hemodynamically stable (BP 100/70 mmHg, HR 70 bpm). ECG showed a normal sinus rhythm. He was treated with IV fluids, atropine (1 mg), and antiemetics. Following 12 h of observation and stabilization, he was discharged without complications.

### 2.3. Case 3

A 66‐year‐old male with chronic obstructive pulmonary disease (COPD) presented with generalized pruritus and facial angioedema after ingesting 5 mL of wild honey. Unlike the typical presentation, he exhibited sinus tachycardia (132 bpm) rather than bradycardia, alongside hypoxia (SpO_2_ 74%) and tachypnea. He was diagnosed with anaphylaxis based on the acute onset of mucosal involvement and respiratory compromise. Immediate management included IV fluids, intramuscular (IM) adrenaline (0.5 mg of 1:1000 solution), hydrocortisone, and antihistamines. He was admitted to the ward due to persistent respiratory distress. He improved over 24 h, and a discharge ECG showed resolution of tachycardia.

### 2.4. Case 4

A 62‐year‐old male presented with abdominal cramps, loose stools, and vomiting after consuming 25 mL of wild honey for a common cold. He presented in shock with unrecordable blood pressure and severe bradycardia (40 bpm). ECG confirmed sinus bradycardia. He required aggressive resuscitation with IV fluids and atropine (2 mg). Labs were within normal limits. He achieved hemodynamic stability and was discharged after 48 h of observation with a normal sinus rhythm.

### 2.5. Case 5

A 56‐year‐old male presented with shortness of breath, palpitations, and weakness after ingesting 10 mL of wild honey. Vital signs were stable (BP: 100/80 mmHg, HR: 78 bpm), and the ECG showed a normal sinus rhythm. He was managed symptomatically with IV fluids and antiemetics. He was observed for 12 h and discharged without residual symptoms.

### 2.6. Case 6

A 41‐year‐old male presented with shortness of breath, generalized urticaria, and pruritus following the ingestion of 15 mL of wild honey for sexual dysfunction. He exhibited signs of anaphylaxis, including respiratory distress (SpO_2_ 74%) and tachycardia (96 bpm), without the classic bradycardia associated with grayanotoxin toxicity. Management included immediate IM adrenaline (0.5 mg of 1:1000 solution), IV fluids, and corticosteroids. He was monitored for 24 h until stable and discharged with a normal sinus rhythm.

### 2.7. Case 7

A 48‐year‐old male with hypertension presented with dizziness and vomiting after accidentally ingesting 20 mL of wild honey. He was hypotensive with unrecordable blood pressure and profound bradycardia (38 bpm). ECG revealed a nodal rhythm. He was treated with atropine (2 mg), IV fluids, and oxygen. Hemodynamic stability was achieved over 48 h, and his rhythm converted to a normal sinus rhythm upon discharge.

The summary of all cases is given in Table 1.

**TABLE 1:** Summary of all cases.

Cases	Case 1	Case 2	Case 3	Case 4	Case 5	Case 6	Case 7
Date of presentation	June 13th, 2024	June 18th, 2024	October 23rd, 2024	December 11th, 2024	December 16th, 2024	December 25th, 2024	January 31st, 2025
Age (in years)	36	39	66	62	56	41	48
Sex	Male	Male	Male	Male	Male	Male	Male
Preexisting comorbidities	None	Hypertension	COPD	None	None	None	Hypertension
Consumed honey for	Recreation	Hypertension	Abdominal pain	Common cold	Common cold	Sexual dysfunction	Accidental exposure
Amount (in mL)	15	10	5	25	10	15	20
Symptoms	Shortness of breath, abdominal cramps, vomiting	Dizziness, vomiting	Swelling of the face, itching of the whole body	Abdominal cramps, loose stool, vomiting	Shortness of breath, palpitation, generalized weakness	Itching, urticaria over the whole body, shortness of breath	Dizziness, shortness of breath, vomiting
Blood pressure at presentation (mmHg)	80/60	100/70	100/60	Unrecordable	100/80	100/70	Unrecordable
Pulse rate (in beats per minute)	48	70	132	40	78	96	38
Signs of anaphylaxis	No	No	Yes	No	No	Yes	No
ECG findings	Sinus bradycardia	Normal sinus rhythm	Sinus tachycardia	Sinus bradycardia	Normal sinus rhythm	Normal sinus rhythm	Nodal rhythm
Management in ER/Ward/ICU	ER	ER	Ward	Ward	ER	Ward	ER
Medication: atropine (mg)	2	1	No	2 mg	No	No	2
Medication: Adrenaline	No	No	0.5 mg (0.5 mL of 1:1000 solution) IM STAT	No	No	0.5 mg (0.5 mL of 1:1000 solution) IM STAT	No
Oxygen requirement	Yes	No	Yes	Yes	No	Yes	Yes
Observation time (in hours)	24	12	24	48	12	24	48
Complications	None	None	None	None	None	None	None
Disposition	Discharged	Discharged	Discharged	Discharged	Discharged	Discharged	Discharged

## 3. Discussion

This case series documents the clinical spectrum of mad honey poisoning, reinforcing known cardiovascular effects while highlighting rare anaphylactic presentations. Mad honey differs from commercial honey due to contamination with grayanotoxins, toxic diterpenes that cause intoxication and poisoning effects [1]. These toxins, also referred to as rhodotoxins, are produced when honey bees extract nectar and pollen from *Rhododendron* species, specifically *R. luteum, R. flavum, R. simsii,* and *R. ponticum* [3]. The traditional practice of hunting and consuming wild honey in the Himalayan regions significantly raises the risk of grayanotoxin exposure [4].

Consistent with previous literature, all patients in this series were male, ranging in age from 36 to 66 years. Most reported cases involve males aged 40–60 [1, 7], likely due to the higher usage of mad honey as a sexual stimulant and the prevalence of hypertension in this demographic [7]. The quantity consumed in our series (5–25 mL) aligns with established toxic thresholds; consumption of approximately 15–30 g of mad honey can cause intoxication, with symptoms appearing within half an hour to four hours [2].

Pathophysiologically, grayanotoxins act on sodium ion channels at the cellular level, modifying their functions by interfering with action potential transmission [8, 9]. The toxins bind to voltage‐dependent sodium channels in their open state, preventing inactivation and leading to prolonged depolarization [10, 11]. This mimics cholinergic agents, resulting in the classic triad of bradycardia, hypotension, and respiratory depression [10, 11]. In our series, significant bradycardia and hypotension were observed in three cases (Cases 1, 4, and 7), necessitating 2 mg of atropine, while Case 2 received 1 mg. Arrhythmias such as atrioventricular block, nodal rhythms, and sinus bradycardia are common [9, 12]. Case 7 notably presented with a nodal rhythm, which resolved upon recovery.

A distinct finding in this series is the presence of anaphylaxis in two patients (Cases 3 and 6). Anaphylaxis has been reported in rare instances [13, 14], yet the mechanism remains distinct from the direct cardiotoxic effects of grayanotoxin. While grayanotoxins typically induce bradycardia via vagal stimulation [15], our anaphylactic cases presented with tachycardia (Case 3) or relative tachycardia (Case 6) and cutaneous signs (urticaria and angioedema). This may suggest a potential IgE‐mediated hypersensitivity to bee proteins or pollen allergens within the honey, rather than a direct toxic effect of the grayanotoxin itself, although further immunological studies are required to establish this definitively. Clinicians must distinguish between the cholinergic crisis of grayanotoxin poisoning (requiring atropine) and anaphylaxis (requiring adrenaline), as the management algorithms differ significantly.

Diagnosis of mad honey intoxication is clinical, based on the history of honey consumption and the presence of unexplained bradycardia and hypotension [7]. It is essential to differentiate this from organophosphate poisoning, which presents with similar cholinergic symptoms. While serum cholinesterase levels can differentiate the two [16], this testing was unavailable in our center; thus, detailed history taking was paramount.

Treatment is largely supportive. Saline infusion acts as the first line for hypotension, with atropine (0.5–2 mg) reserved for severe bradycardia [2, 9]. In our series, all patients responded well to conservative management and pharmacological intervention where indicated. The prognosis is generally excellent [17], with symptoms typically resolving within 24 h [6].

Limitations of this study include its design as a small case series from a single center and the lack of laboratory confirmation of grayanotoxin levels in the honey or patient serum. Anaphylaxis was diagnosed based on the clinical presentation, and the rapid response to adrenaline strongly supports the diagnosis.

## 4. Conclusion

This case series highlights the potential risks associated with the consumption of wild honey contaminated with grayanotoxins. It underscores the importance of public awareness about the risks associated with traditional practices involving wild honey, particularly in regions where mad honey is common. Healthcare providers should consider mad honey intoxication in patients with unexplained bradycardia and hypotension, and a thorough history of mad honey exposure can help differentiate it from organophosphate poisoning, which presents with similar symptoms and is commonly encountered in countries such as Nepal. This paper contributes to the growing body of literature on mad honey poisoning, emphasizing the need for further research and education to prevent similar incidents.

## Author Contributions

A.B. and K.K.C. prepared and edited the entire manuscript.

## Funding

No funding was received for this research.

## Ethics Statement

Ethical approval was exempted by the Institutional Review Board, as institutional policy does not require formal ethical approval for anonymized retrospective case series.

## Consent

Written informed consent was obtained from all the patients for the publication of this case series. A copy of written consent is available for review by the editor‐in‐chief of this journal on request.

## Conflicts of Interest

The authors declare no conflicts of interest.

## Data Availability

The data that support the findings of this study are available from the corresponding author upon reasonable request.
